# Responsibilities or Requirements: Framing Dual Use Issues for Scientific Engagement

**DOI:** 10.3389/fpubh.2014.00107

**Published:** 2014-08-11

**Authors:** Jo L. Husbands

**Affiliations:** ^1^Board on Life Sciences, U.S. National Academy of Sciences, Washington, DC, USA

**Keywords:** biosecurity, bioterrorism, dual use, responsible science, non-proliferation

## Introduction

Efforts to prevent the proliferation of biological weapons or bioterrorism face an increasingly complicated landscape characterized by rapid scientific and technological progress, growing global diffusion of research capacity, and an array of stakeholders with important potential roles. In 2002, the International Committee of the Red Cross (ICRC) introduced the concept of a “web of prevention” to underscore the need for a comprehensive and coordinated strategy that could engage gather the many communities necessary to address the challenges (1). The scientific community, broadly defined to encompass the many fields beyond biology that now make up the life sciences research enterprise, is recognized as essential to the success of any strategy. This is particularly true for addressing what has come to be called “dual use” research, which is undertaken for beneficial purposes but has the potential to be misused to cause deliberate harm (2–5). Programs for “scientific engagement” have expanded in the last decade to reflect this recognition and policy attention.

## Approaches to Engagement: Framing the Issues

Approaches to engaging scientists in biosecurity issues generally follow one of two approaches. The traditional framing starts with *requirements*, the legal obligations to which scientists are subject, broadly under international treaties as well as specifically under the national laws and regulations that either implement the agreements or are undertaken independently by countries for their own security purposes (6). In cases such as the European Union, scientists may also be subject to a significant regional regulatory framework. It should be no surprise that the natural inclination for those in the security and law enforcement communities, as well as for the diplomats who tend the treaties, is to begin with one’s legal obligations, what a scientist “must” do.

An alternative framing that appears to be gaining momentum treats biosecurity within the broader context of the social responsibility of science as another example of the *responsibilities* that scientists are expected to fulfill. Science is not conducted in a social vacuum and scientists are subject to the effects of many broader forces (7). Among them, changing social attitudes clearly affect how science is carried out^1^. What scientists “should” do thus comes from norms of professional behavior as much, or in some cases perhaps more, as from legal requirements (8). It also allows scientific engagement on biosecurity to take advantage of the international attention to issues of research integrity and responsible conduct of science. This growing attention reflects the need for common understandings as the life sciences have become an increasing global enterprise^2^. High-level declarations and statements have underscored the ethical imperative that along with the fundamental principles of freedom in the conduct of science come responsibilities and the need to maintain public trust^3^.

An example of nesting security within the broader framing comes from a project of the InterAcademy Council (IAC) and IAP – The Global Network of Science Academies^4^. In its first phase, an international committee formed by the IAC and IAP produced a short policy report on research integrity (9). The report addresses a broad range of issues, including security. The report notes, for example, that Science and other forms of scholarship have been incredibly productive by seeking knowledge unfettered by tradition, ideology, and external pressure. At the same time, research can have a profound influence on the environment, human health and well-being, economic development, national security, and many other facets of human life. Many areas of science and technology can be used for destructive as well as constructive purposes and researchers have a special responsibility to understand and address issues of “dual use.” Research on biological pathogens, for example, poses both risks and benefits for human health [Ref. (9): p. 15].

The report then concludes that “researchers should bear in mind the possible consequences of their work, including harmful consequences, in planning research projects” [Ref. (9): p. 16], which has clear implications for scientists’ roles in addressing dual use issues.

## Conclusion

The two approaches to framing scientific engagement on biosecurity are not mutually exclusive. Many laws reflect social norms and science engagement programs using a framework of responsible science include discussions of laws and regulations, with the Biological Weapons Convention as the international legal embodiment of a fundamental norm against using disease as a weapon. And much more needs to be done to decide on and develop the appropriate mix of legal, regulatory, and policy measures to address the security challenges posed by globalizing science. The issue is where to begin and what works best to reach one group of essential stakeholders. Responsible conduct offers a foundation on which one can build and complements more detailed attention to security issues and legal requirements needed by those in certain areas of research. It can also contribute to making scientists part of the solution to biosecurity challenges rather than part of the problem.

## Conflict of Interest Statement

The author declares that the research was conducted in the absence of any commercial or financial relationships that could be construed as a potential conflict of interest.

## Footnotes

^1^A clear example is the development of standards for the treatment of human subjects in experiments, which developed over time, particularly during the twentieth century in response to egregious abuses by researchers. The standards for the treatment of laboratory animals have continued to evolve as well.

^2^For example, the Global Research Council, created in 2012, is a virtual organization comprises national science and engineering funding bodies from about 50 countries that is devoted to promoting high quality research collaborations, including issues of research integrity. More information is available at http://www.globalresearchcouncil.org/.

^3^A discussion of these developments may be found in a report from the National Research Council (10).

^4^IAP is a global network of more than 100 of the World’s Science Academies, launched in 1993. Its primary goal is to help member academies work together to advise citizens and public officials on the scientific aspects of critical global issues. More information is available at http://www.interacademies.net/. The IAC produces reports on scientific, technological, and health issues related to the great global challenges of our time to provide knowledge and advice to national governments and international organizations. More information is available at http://www.interacademycouncil.net/
